# Could a brief assessment of negative emotions and self-esteem identify adolescents at current and future risk of self-harm in the community? A prospective cohort analysis

**DOI:** 10.1186/1471-2458-13-604

**Published:** 2013-06-22

**Authors:** Rhiannon Phillips, Melissa R Spears, Alan A Montgomery, Abigail Millings, Kapil Sayal, Paul Stallard

**Affiliations:** 1 Department for Health, University of Bath, 22-23 Eastwood, Claverton Down, Bath BA2 7AY, UK; 2 School of Social and Community Medicine, University of Bristol, 39 Whatley Road, Bristol BS8 2PS, UK; 3 Nottingham Clinical Trials Unit, Nottingham Health Science Partners, C Floor, Queen’s Medical Centre, South Block, Nottingham NG7 2UH, UK; 4 Department of Psychology, University of Sheffield, Western Bank, Sheffield S10 2TP, UK; 5 Division of Psychiatry and Institute of Mental Health, University of Nottingham, Nottingham NG7 2UH, UK; 6 Institute of Primary Care & Public Health, Cardiff University, School of Medicine, Heath Park, Neuadd Meirionnydd, Cardiff CF14 4YS, UK

**Keywords:** Self-harm, Screening, Adolescents, Negative emotions, Self-esteem

## Abstract

**Background:**

Self-harm is common in adolescents, but it is often unreported and undetected. Available screening tools typically ask directly about self-harm and suicidal ideation. Although in an ideal world, direct enquiry and open discussion around self-harm would be advocated, non-psychiatric professionals in community settings are often reluctant to ask about this directly and disclosure can be met with feeling of intense anxiety. Training non-specialist staff to directly ask about self-harm has limited effects suggesting that alternative approaches are required. This study investigated whether a targeted analysis of negative emotions and self-esteem could identify young adolescents at risk of self-harm in community settings.

**Methods:**

Data were collected as part of a clinical trial from young people in school years 8–11 (aged 12–16) at eight UK secondary schools (N = 4503 at baseline, N = 3263 in prospective analysis). The Short Mood and Feelings Questionnaire, Revised Child Anxiety and Depression Scale, Rosenberg Self-Esteem Scale, personal failure (Children’s Automatic Thoughts Scale), and two items on self-harm were completed at baseline, 6 and 12 months.

**Results:**

Following a process of Principal Components Analysis, item reduction, and logistic regression analysis, three internally reliable factors were identified from the original measures that were independently associated with current and future self-harm; personal failure (3 items), physical symptoms of depression/anxiety (6 items), positive self-esteem (5 items). The summed score of these 14 items had good accuracy in identifying current self-harm (AUC 0.87 girls, 0.81 boys) and at six months for girls (0.81), and fair accuracy at six months for boys (AUC 0.74) and 12 months for girls (AUC 0.77).

**Conclusions:**

A brief and targeted assessment of negative emotions and self-esteem, focusing on factors that are strongly associated with current and future self-harm, could potentially be used to help identify adolescents who are at risk in community settings. Further research should assess the psychometric properties of the items identified and test this approach in more diverse community contexts.

## Background

Self-harm in adolescents and young adults represents an important public health issue [1]. Community surveys indicate that around 5 to 10% of adolescents report self-harm over the last year [2-5]. International comparisons for 15 to16 year olds have indicated that rates of self-harm in the UK are amongst the highest in developed countries, with 3.2% of males and 11.1% of females reporting self-harm over the last year and with lifetime prevalence rates of 4.8% for males and 16.7% for females [3]. Despite its high prevalence, self-harm in adolescents often goes unreported and undetected [3]. As previous self-harm increases the risk of doing so again and repeated self-harm is a risk factor for suicide [6-9], proactive identification of young people who are at risk is important.

Self-harm is referred to in several ways in the literature, including ‘self-mutilation’, ‘non-suicidal self-injury’ (NSSI), ‘self-injurious behavior’, ‘parasuicide’, ‘self-wounding’, or ‘self-poisoning’ [10]. The most common methods of self-harm reported in community settings are self-cutting (or self-laceration) and self-battery (e.g. head-butting a wall or pulling hair) [10-12]. Self-poisoning (or overdose) is less common in the community, but is strongly associated with the presence of suicidal intent [11] and is the most common method in those presenting to hospital following self-harm [13]. Self-poisoning is more common in girls than in boys, who more frequently report self-battery as a method of self-harm [11,12]. Motivations commonly reported for self-harm include: coping with negative emotions; self-loathing; anger; self-punishment; loneliness; distraction from problems, and; to communicate bad feelings to others [11,12]. Girls are more likely to report reducing negative emotions as a motivator, while boys have a greater tendency to report more superficial reasons like boredom or curiosity [11,12]. Almost half of young people report feeling better after self-harming and this is most common in those who self-harm frequently [11]. However, feelings of guilt, shame, and disgust can also increase following self-harm [12].

While there are key differences between self-harm with and without suicidal intent in terms of different methods of self-harm, motivations, reinforcers, neurobiology, and association with suicide, they also share some common risk factors and can occur in the same individuals [7,14,15]. Approximately 25% of adolescents who have self-harmed report having suicidal intent during their last episode [11]. Kidger et al. [11] state that:

“Although the majority of self-harm behaviour is not accompanied by a desire to die, all self harm regardless of motivation is associated with increased risk of suicidal thoughts and plans, particularly when it is carried out repeatedly” (p. 1).

Therefore, while there are various definitions of self-harm, this manuscript adopts a broad definition to encompass deliberate self-injury or self-poisoning, in line with British guidelines [16], and includes self-harm with or without suicidal intent.

A wide variety of assessments have been developed that directly inquire about self-harm and suicidal ideation in adolescents, including the Columbia Suicide Screen, Suicide Risk Screen, and the Risk-Taking and Self-Harm Inventory for Adolescents [17-19]. However, self-harm and suicide are sensitive and stigmatised issues. Non-mental health specialists are not typically accurate in identifying mental health problems (particularly internalizing disorders) and can find it difficult to distinguish between normal variation in mood and precursors to more serious mental health problems [20-23]. People who are not mental health professionals, such as teachers and youth justice workers, find it difficult to ask adolescents about suicide and self-harm and disclosure can be met with feelings of intense anxiety [24-26]. There is also a pervasive concern that asking about suicidal thoughts or behaviour could trigger suicidal ideation or attempts, despite evidence that enquiring about suicide is not harmful [27].

The reluctance of non-psychiatric professionals to directly ask about self-harm has led some to investigate whether training community-based professionals can increase awareness and improve identification. However, training school-based staff has variable results and seems to particularly benefit those who are already able to talk with students about suicide and distress [28]. Whilst helping non-psychiatric professionals to talk about self-harm would be the ideal solution, practically they find this very difficult and alternative more indirect approaches need to be investigated.

A number of risk factors for self-harm have been identified including depressed mood, increased anxiety, low self-esteem and cognitions that focus upon self-failure [1,29-32]. Depression and anxiety in adolescence are associated with an increased incidence of self-harm in young adulthood [4]. Self-report measures can assess these variables in adolescents in community settings in a valid and reliable way [33-36]. An indirect approach such as this would be more acceptable and offers the potential to identify those who are self-harming or at increased risk of future self-harm. However, general measures of depression and anxiety may lack discriminative ability in distinguishing between those who do and do not self-harm [10]. The aims of this study are to investigate whether a brief set of items can be identified from existing measures of negative emotion and self-esteem that are sufficiently sensitive and specific to identify adolescents at risk of self-harm in community settings.

## Methods

### Design

These prospective cohort data were obtained during a multi-centre cluster randomised controlled trial [37,38]. Assessments took place at baseline, six and 12 months. Self-report questionnaires were completed anonymously at school in sessions led by the research team.

### Setting and participants

Eight non-denominational mixed-sex secondary schools in the South West and East Midlands in England took part in the study between 2009 and 2011. A total of 5030 young people consented to participate in the trial (91.5% of the eligible population), with N = 4140 (86.5%) retained at 12 month follow-up [38]. Participants who had completed baseline self-harm measures were included in our cross-sectional psychometric analysis (N = 4503), and those with complete self-harm data at all three time points were included in the prospective cohort analysis (N = 3263). All pupils in Years 8–11 (aged 12–16 years) in participating schools were eligible, unless they were not attending school (e.g. due to long term sickness, being excluded from school) or did not participate in Personal Social and Health Education (PSHE) lessons for religious or other reasons.

Participation required written consent from the school head teacher, parental consent on an opt-out basis, and written assent from the adolescent. A safety procedure was in place to inform young people and their parents by letter to their home address if they scored highly on the primary outcome measure for the trial (symptoms of depression assessed by the Short Form-Mood and Feelings Questionnaire) to signpost them to relevant services should they wish to seek support/advice. All young people were given a printed list of sources of support should they have any concerns during each assessment session. There was also a written adverse events procedure approved by the Data Monitoring and Ethics Committee (DMEC) in place as part of the trial. The study was approved by the University of Bath School for Health ethics committee.

### Measures

#### Primary outcome: self-harm

The self-harm questions were adapted from those used in the Avon Longitudinal Study of Parents and Children [39]. The ALSPAC study included a detailed survey of self-harm in n = 4810 young people who had been followed since birth at age 16–17 years [11]. The wording of the item relating to self-harm acts used by Kidger et al. [11] was based on Childhood Interview for DSM-IV Borderline Personality Disorder (CI-BDP) question asked during clinic interviews with the ALSPAC sample at age 11 [40]. A detailed assessment of self-harm motivation and methods was not possible in the current study as this was part of a wider assessment of mental health and related issues carried out as part of a clinical trial. We therefore focused on two key issues; whether young people had harmed themselves deliberately in the last six months, and whether they had thought about harming themselves (even if they had not done so). Furthermore, we were interested in relatively recent, rather than lifetime, prevalence of self-harm to help identify those who were currently at risk or may be in the near future.

Self-harm acts were therefore assessed using a single item at baseline, six and 12 months;

“Have you ever hurt yourself on purpose in any way (e.g. by taking an overdose of pills or by cutting yourself) in the last 6 months?”

This was rated on a 3-point scale (0 – not at all, 1- Once, 2 – 2 or more times) but for analysis was coded as a binary outcome (never vs. ever in the last six months).

#### Self-harm thoughts

As with acts of self-harm, thoughts about self-harm (even if an act had not taken place) were also assessed at each time point using a single item;

“Have you thought about hurting yourself, even if you would not really do it, in the last 6 months?”

This was rated on a 3-point scale (0 – not at all, 1- Once, 2 – 2 or more times) but for analysis was coded as a binary outcome (never vs. ever in the last six months).

#### Short mood and feelings questionnaire (SMFQ) [33]

This 13 item questionnaire assesses symptoms of low mood. Respondents rate each item as ‘not true’ (0), ‘sometimes’ (1), or ‘true’ (2), with scores summed to provide a total score (range = 0–26). The SMFQ has been used in community samples, correlates well with other measures of depression, has good test/re-test reliability, and higher scores tend to be associated with fulfilling diagnostic criteria for clinical depression [33,41].

#### Children’s automatic thoughts scale (CATS; personal failure subscale) [36]

The CATS was developed to assess the automatic negative thoughts that children have which are associated with psychiatric complaints. It has been validated in clinical and community settings [42]. We used the 10 item personal failure sub-scale as this is the most closely associated with depression and self-blame. Items are rated from ‘not at all’ (0) to ‘all the time’ (4). It has high internal reliability (alpha 0.92), acceptable test-retest reliability (0.74), and differentiates between clinically depressed and anxious young people and a community group [36].

### The Rosenberg self-esteem scale (RSE) [35]

Assessing levels of self-worth and self-acceptance, this scale consists of 10 statements answered on a four-point scale, ranging from ‘strongly disagree’ (0) to ‘strongly agree’ (3). Scores for the positive self-esteem items are reversed. It has demonstrated good reliability and validity across different sample groups and has been validated for use with adolescents [35,43].

### The revised child anxiety and depression scale – 25 item version (RCADS-25) [34]

The RCADS-25 assesses changes in symptoms of DSM-defined anxiety disorders and major depression in children. Five sub-scales assess symptoms of generalised anxiety disorder, separation anxiety disorder, social phobia, panic disorder and major depressive disorder. Items are rated on a four point scale, from ‘never’ (0) to ‘always’ (3). The RCADS-25 is comparable to the full length version in terms of reliability, internal consistency, test–retest stability, and it has reasonable parent–child agreement and good convergent and divergent validity [34].

### Demographics

Data were gathered on age, gender, ethnicity, and household composition.

### Analysis

Statistical analysis was carried out using Stata (Version 12). Our approach to analysis was firstly to establish to what extent the measures we included in the study represented distinct factors, given the likely level of inter-correlation between the scales. As an assessment to identify young people at risk of self-harm in the community would need to be brief, we went through a process of item reduction. The association between the reduced factors and current and future self-harm was then examined. Finally, we assessed how accurate these factors were in identifying young people who reported self-harm.

#### Stage 1: item reduction and psychometric analysis

The original measures were reduced using exploratory factor analysis (Principal Components Analysis method). Scaling varied slightly as the items were derived from different measures (scored 0–2, 0–3, or 0–4), but these were reasonably comparable and there is potential to rescale items for use in future studies. Velicer’s MAP Criteria and parallel analysis were used to determine the appropriate minimum number of factors to retain for rotation. It was anticipated that the measures of negative emotionality would be correlated with each other, so oblique rotation was applied to facilitate interpretation [44]. Suitability of data for exploratory factor analysis was checked using the Kaiser-Meyer-Oklin measure of sampling adequacy (overall value = 0.98, all individual item values >0.89).

To produce a measure that was as brief and robust as possible, item reduction involved removing items not loading highly onto any factor (<0.5), followed by a process of removing items that were very frequently (>85%) or infrequently (<15%) endorsed and items with least variance within each scale to ensure sufficient variation in responses within a non-clinical population [45,46]. The aim of this process was to obtain the simplest factor structure with fewest items, whilst maintaining a sufficiently high level of reliability for each subscale (Cronbach’s alpha >0.8). Correlations between the new and original measures were examined to assess convergent validity.

#### Stage 2: logistic regression to examine associations between the reduced measures and current and future self-harm

Sensitivity analysis investigated clustering within the data (individual, class, year group, and school) using multi-level logistic regression models. However, inclusion of these levels made no material difference to estimated associations or standard errors, and therefore simple logistic regression models were used. Analyses were conducted separately for males and females, as both common and distinct factors associated with self-harm have been identified for girls and boys [1]. Age was included in all models.

#### Accuracy in identifying young people who self-harm

Receiver Operating Characteristics (ROC) analysis was carried out for self-harm at each time point, where sensitivity is plotted against (1 – specificity) and Area Under the Curve (AUC) values were calculated. Sensitivity, specificity and% correctly classified of the total scale and subscale scores were examined to identify the optimal cut-off points, with sensitivity being given priority. Analysis was carried out separately for males and females to ensure cut-off points were gender appropriate.

## Results

### Sample characteristics

2275 males (50.5%) and 2228 females (49.5%) with a mean age in years of 14.0 (SD 1.1) completed the baseline self-harm measure and were included in the cross-sectional psychometric analysis. They were predominantly Caucasian (85.6%) and the majority lived with both parents (65.5%). Self-harm over the last six months was reported by 432 (9.6%) participants. Females were more likely than males to report self-harm (OR 1.90, 95% CI 1.55-2.34).

The self-harm outcomes at all three time points were completed by 1631 boys and 1632 girls, who were included in the prospective analysis. Participant characteristics for the prospective analysis were similar to those included in the cross-sectional analysis; 114 (7%) boys and 180 (11%) girls reported self-harm acts at baseline. Similar prevalence was reported at 6 months (8% of boys, 12% of girls) and 12 months (7% of boys, 13% of girls). Data completeness was good, with <10% of scale totals or individual items missing. Where fewer than 20% of items were missing on a scale, these items were replaced with the mean. Otherwise, the individual’s score was coded as missing. This was appropriate as there was little missing data, and imputation has been shown to make little difference in studies of quality of life studies under these conditions [47]. Those with complete self-harm data at all three time points were on average younger (mean age 13.8 vs. 14.4), reported less depression (mean SMFQ 4.0 vs. 5.1) and anxiety (mean 11.9 vs. 14.4), had higher self-esteem (mean 21.5 vs. 20.7), and were less likely to report self-harm acts at baseline (9.0% vs. 11.3%) than those with missing self-harm data.

### Item reduction

Five interpretable factors were identified via Principal Components Analysis; personal failure, generalised anxiety, physical symptoms of depression/anxiety, positive self-esteem and separation anxiety. Separation anxiety (3 items) was removed from further analysis as the scale had low reliability (alpha = 0.59). From the remaining four factors, 19 items were removed due to low factor loadings (<0.5), 4 items were removed as they were infrequently endorsed (<15%) and therefore there was insufficient variation in responses within the population, and a further 15 items were removed on the basis that they had the least impact on scale variance if removed. This process left a total of 19 items loading on to four distinct, interpretable, and internally reliable factors (Table 1).

**Table 1:** Factor loadings of the 19 items retained in the reduced factors

**Factor**	**Question wording & items**	**Original scale**	**Factor loading**
**Personal failure**	Nothing ever works out for me anymore	CATS	0.57
	It’s my fault that things have gone wrong	CATS	0.62
	I’ve made such a mess of my life	CATS	0.77
**Generalised anxiety**	I worry about bad things happening to me	RCADS	0.74
	I worry about making mistakes	RCADS	0.69
	I am worried that I will do badly at school work	RCADS	0.70
	I worry about doing poorly at things	RCADS	0.75
	I worry about what will happen	RCADS	0.73
**Physical symptoms of anxiety and depression**	My heart suddenly beats too quickly for no reason	RCADS	0.63
	I am tired a lot	RCADS	0.74
	I feel like I don’t want to move	RCADS	0.69
	I felt so tired I just sat around and did nothing	SMFQ	0.69
	I was very restless	SMFQ	0.65
	I found it hard to think or concentrate properly	SMFQ	0.54
**Positive self-esteem**	On the whole, I am satisfied with myself	RSE	0.64
	I take a positive attitude towards myself	RSE	0.66
	I feel that I have a number of good qualities	RSE	0.85
	I am able to do things as well as most other people	RSE	0.77
	I feel that I am a person of worth, at least as equal as others	RSE	0.72

A summary of factor characteristics and correlations with the original related measures are provided in Table 2. Due to the nature of the items included, some had skewed rather than normal distributions. Exploratory Factor Analysis is relatively robust to violation of the normality assumption [48]. Log-transforming and standardising the items made no difference to the factor structure.

**Table 2:** Scale characteristics of the factors retained following Principal Components Analysis

**Factor**	**Number of items**	**Alpha**	**Scale mean**	**Scale SD**	**Range of summed total**	**Pearson’s correlation with related original measures**	
Total score (personal failure, physical symptoms of anxiety/depression and positive self-esteem)	14	0.87	9.06	6.44	0-42	SMFQ	0.80
						RCADS total	0.78
						CATS personal failure	0.75
							−0.81
						RSE	
Personal failure	3	0.86	1.72	2.53	0-12	CATS personal failure	0.93
Generalised anxiety	5	0.83	3.79	2.88	0-15	RCADS generalised anxiety disorder	0.79
Physical symptoms of anxiety and depression	6	0.82	2.95	2.97	0-15	RCADS depression	0.88
							0.72
						RCADS panic	0.81
						SMFQ	
Positive self-esteem	5	0.82	10.51	2.66	0-15	RSE	0.86

### Logistic regression models

Adjusted independent effects of reduced factors in the regression models are provided in Table 3. For the four reduced factors the Variance Inflation Factor (VIF) ranged from 1.42 to 2 in the cross-sectional analysis, with a mean VIF of 1.79, indicating that multi-collinearity among items was not a problem.

**Table 3:** Self-harm behaviour: odds ratios and 95% confidence intervals in logistic regression

	Males: adjusted* OR (95% CI)	Females: adjusted* OR (95% CI)
**Baseline self-harm**		
Personal failure	**1.18 (1.08 to 1.30)**	**1.24 (1.15 to 1.34)**
Generalised anxiety	1.06 (0.97 to 1.16)	1.02 (0.95 to 1.10)
Physical symptoms	**1.25 (1.15 to 1.35)**	**1.16 (1.07 to 1.24)**
Positive self-esteem	0.95 (0.87 to 1.04)	**0.82 (0.75 to 0.89)**
**6 month self-harm**		
Personal failure	**1.22 (1.12 to1.32)**	**1.19 (1.11 to 1.28)**
Generalised anxiety	1.03 (0.95 to 1.12)	1.03 (0.97 to 1.11)
Physical symptoms	**1.12 (1.04 to 1.21)**	1.14 (1.06 to 1.22)
Positive self-esteem	0.97 (0.90 to 1.06)	**0.87 (0.81 to 0.94)**
**12 month self-harm**		
Personal failure	1.05 (0.95 to 1.16)	**1.16 (1.08 to 1.25)**
Generalised anxiety	1.08 (1.00 to 1.18)	1.01 (0.95 to 1.08)
Physical symptoms	**1.16 (1.07 to 1.25)**	**1.12 (1.05 to 1.2)**
Positive self-esteem	1.01 (0.93 to 1.1)	**0.88 (0.82 to 0.95)**
**Future self-harm models: further adjusted for baseline self-harm thoughts and acts**		
**6 month self-harm**		
Personal failure	**1.13 (1.03 to 1.24)**	1.06 (0.98 to 1.16)
Generalised anxiety	0.98 (0.9 to 1.08)	1.02 (0.95 to 1.1)
Physical symptoms	1.05 (0.96 to 1.14)	1.08 (0.99 to 1.16)
Positive self-esteem	1 (0.92 to 1.1)	**0.91 (0.84 to 0.99)**
Self-harm thoughts	**4.12 (2.44 to 6.97)**	**3.36 (2.13 to 5.29)**
Self-harm acts	**3.58 (2.06 to 6.22)**	**5.8 (3.68 to 9.13)**
**12 month self-harm**		
Personal failure	0.97 (0.87 to 1.07)	1.06 (0.98 to 1.14)
Generalised anxiety	1.05 (0.96 to 1.15)	0.99 (0.93 to 1.06)
Physical symptoms	**1.1 (1.01 to 1.2)**	1.06 (0.99 to 1.15)
Positive self-esteem	1.04 (0.94 to 1.13)	**0.91 (0.84 to 0.98)**
Self-harm thoughts	**2.25 (1.29 to 3.92)**	**3.1 (2.03 to 4.75)**
**Self-harm acts**	**4.69 (2.65 to 8.3)**	**2.91 (1.86 to 4.55)**

*All models adjusted for age. NB: All scales within each model were entered simultaneously and therefore OR and CIs are for independent effects of each scale. Where there is strong evidence of association, this is highlighted in bold text.

For girls, personal failure, physical symptoms of anxiety and depression, and low positive self-esteem were associated with self-harm at all time points. For boys, personal failure was associated with self-harm at baseline and six months, while physical symptoms of anxiety/depression were associated with self-harm at all three time points. Positive self-esteem was not independently associated with self-harm at any time point for boys, suggesting that this played a more important role for girls. Self-harm and thoughts of self-harm at baseline were strongly associated with self-harm at follow-up (see Table 3). After adjustment for these in the models, personal failure (6 months) and physical symptoms of anxiety and depression (12 months) for boys, and low positive self-esteem for girls (6 and 12 months) remained independently associated with future self-harm. Generalised anxiety was not associated with self-harm in girls or boys at any time point and was excluded from further analysis.

### Identification of adolescents who self-harm

The final analysis consisted of 14 items from the original measure, comprising three subscales (personal failure, physical symptoms of depression/anxiety, and positive self-esteem – see Table 1 for individual items in each factor). The AUC values, sensitivity, and specificity of the summed score of these items for two points on either side of the suggested cut-off points are shown in Table 4.

**Table 4:** Total score of the 14 items at baseline: area under the ROC Curve (AUC), sensitivity and specificity in identifying current and future self-harm

	**Males**						**Females**					
	**AUC**	Cut-off ^*^	% scoring above cut-off ^*^	**Sens (%)**	**Spec** (**%)**	**Corr class (%)**	**AUC**	Cut-off ^*^	% scoring above cut-off ^*^	**Sens (%)**	**Spec (%)**	**Corr class (%)**
**Current self-harm**	**0.81**						**0.87**					
		≥7	40.9	85.71	56.91	58.91		≥10	34.7	90.56	66.04	68.77
		≥8	33.9	83.93	63.96	65.35		≥11	29.7	87.22	71.88	73.58
*Suggested cut*-*off*		**≥9**	**27.1**	**77.68**	**71.08**	**71.53**		**≥12**	**25.7**	**82.22**	**76.39**	**77.04**
		≥10	18.2	72.32	77.39	77.04		≥13	22.7	77.22	80.35	80.00
		≥11	14.7	67.86	82.05	81.06		≥14	19.7	76.11	82.99	82.22
**6 month self-harm**	**0.74**						**0.81**					
		≥7		79.84	56.77	58.54		≥10		80.50	65.42	67.28
		≥8		73.39	63.40	64.17		≥11		76.00	71.13	71.13
		≥9		67.74	70.64	70.42		≥12		76.00	71.13	71.73
		≥10		61.29	76.68	75.50		≥13		68.50	79.93	78.52
		≥11		57.26	81.57	79.70		≥14		67.00	82.61	80.68
**12 month self-harm**	**0.68**						**0.77**					
		≥7		67.77	55.72	56.62		≥10		76.47	64.97	66.42
		≥8		61.98	62.47	62.44		≥11		72.06	70.69	70.86
		≥9		57.02	69.70	68.75		≥12		67.65	75.28	74.32
		≥10		53.72	76.19	74.50		≥13		60.78	78.88	76.67
		≥11		48.76	80.80	78.40		≥14		56.86	81.29	78.21

^*^Cut-offs refer to total scores at baseline and their ability to identify those currently reporting self-harm or doing so 6 and 12 months later. Data shown for two points around the suggested baseline cut-off point for girls (≥12) and boys (≥9). *Sens*, Sensitivity, *Spec*, Specificity, *Corr class*, Correctly classified.

**Figure 1:**
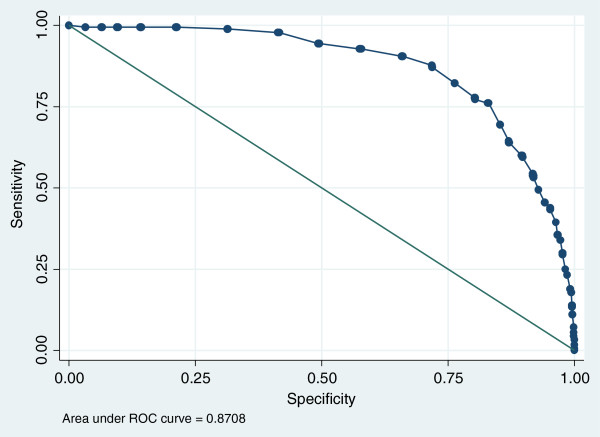
Baseline ROC curve for the total score of the final 14 items and self-harm in girls.

**Figure 2:**
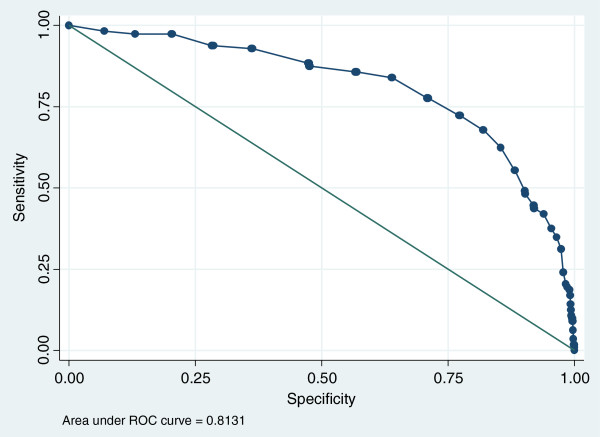
Baseline ROC curve for of the final 14 items and self-harm in boys.

While the AUCs for individual subscales ranged from 0.70 to 0.81 for boys and 0.79 to 0.84 for girls (see Additional file 1), the highest level of screening accuracy was achieved using the summed total score of the 14 items. This produced high AUCs for girls (0.87) and boys (0.81) at baseline. At follow up, the AUC for girls was still above 0.8 at six months and is within the ‘fair’ range (0.77) at 12 months. For boys, accuracy was fair at 6 months (AUC 0.74) but fell below acceptable levels by 12 months (Figures 1 and 2).

## Discussion

### Principal findings

This study set out to examine whether particular items from existing widely used measures of negative emotions and self-esteem could help identify adolescents at risk of self-harm in a community setting. From the original measures, we were able to identify a set of 14 items which comprised three distinct factors; personal failure (3 items), physical symptoms of depression/anxiety (6 items) and positive self-esteem (5 items). All three factors had good internal reliability (α > 0.8), good convergent validity with the original measures, and were independently associated with self-harm. The summed score of these 14 items had good accuracy in identifying current self-harm (AUC 0.87 girls, 0.81 boys) and at six months for girls (0.81), and fair accuracy at six months for boys (AUC 0.74) and 12 months for girls (AUC 0.77). These items could therefore potentially be used as a brief assessment of emotional well-being that can specifically and sensitively identify young people at risk of self-harm in a community context.

### Findings in relation to previous research

In line with previous research, this study indicated that negative emotionality, feelings of self-blame, and self-esteem were associated with self-harm [4,30,49]. Our prospective analysis confirmed that these factors were also predictive of future self-harm over the following year. When we further adjusted the regression models for previous self-harm, self-harm acts and thoughts at baseline were strong predictors of self-harm at follow up, indicating that history of self-harm is an important risk factor [6,15]. Nonetheless, in the present study, personal failure (6 months) and physical symptoms of anxiety/depression (12 months) for boys, and low self-esteem for girls (6 and 12 months) remained independently associated with future self-harm when adjusting for previous self-harm thoughts and acts. This type of assessment can therefore add valuable information, even when previous history of self-harm is known.

In terms of gender differences, associations were generally stronger, positive self-esteem appeared to play a more important role, screening accuracy was better, and cut-off points were slightly higher for girls than for boys. This may be because girls are at higher risk of depression and self-harm [3,50]. Boys are also more likely to report high self-esteem than girls [51]. As positive self-esteem may act as a protective factor for self-harm and suicide [52], it may be particularly important to be aware of and promote self-esteem for girls. Nonetheless, it was clear that negative emotionality was an important predictor of self-harm for both genders.

### Implications

Community setting, including primary care and schools, are important locations for improving the mental well-being of young people as they provide access to a wide range of the population and can be a convenient setting for mental health services, voluntary screening, early intervention and prevention programmes [23,53,54]. Adolescents have also emphasized the importance of the school setting in preventing self-harm [55]. While asking directly about self-harm is likely to be the ‘gold standard’ in terms of identifying those at risk in community settings, non-psychiatric professionals working in the community are often reluctant to ask about self-harm even following training [28].

The challenges in identifying young people who self-harm in the community have previously been reported by Ross and Heath [10], who attempted to address this issue by embedding a question about deliberate self-harm in a series of questions about ‘how I deal with stress’. Although this is a promising approach, this still required direct enquiry about self-harm and other potentially sensitive risky behaviours. Further, the authors note the need to employ multiple approaches to assessing self-harm [10].

Our analysis indicates that a tailored assessment of negative thoughts and emotions can provide a potentially reliable, sensitive and specific indicator of who is at risk of self-harm, which could be a convenient and acceptable way of helping to identify those who are at risk in community settings. This is a method worth exploring further given the problems around acceptability of asking directly about self-harm [24-27], and lack of specificity of more general measures of depression or anxiety [10]. Although 14 items remained in our final analysis to identify those at risk of self-harm (which would constitute a longer assessment than asking just one direct question about self-harm), this reduced set of items offers a quick, brief assessment of negative emotions that are strongly associated with self-harm. We do not view this kind of assessment as a way of avoiding discussing self-harm, but rather as part of a toolkit used by non-psychiatric professional to help them feel more confident in recognizing where further enquiry and/or signposting to relevant services may be required. This type of assessment could also potentially be used in conjunction with local knowledge of other risk factors, such as previous self-harm, history of self-harm by friends or family, quality of social relationships, stressful life events, and known history of abuse [3,15,56-58].

Assessing negative emotions and self-esteem in this way allows for identification of young people are at risk of future self-harm, even if they are not currently reporting self-harm thoughts or acts, and could therefore be an useful early onset indicator. This could be have applications in targeting preventative interventions. Given the association between psychological distress in adolescence and self-harm in young adulthood, identifying and addressing common mental health problems in adolescence is likely to be an important component of suicide prevention [4]. The importance of positive self-esteem in girls highlighted in the present study is in line with the view that while treatments for self-harm and depression often focus on negative emotions, improving positive emotional health by enhancing personal and family resources may also be an important aspect of treatment [14]. Eating disorders and self-harm often come hand in hand, with emotional dysregulation being an important feature of both [59]. The potential for using a similar assessment in the context of eating disorders and other related risk behaviours should be explored.

### Strengths and weaknesses

Strengths of this study are that analyses were based on a large sample with high participation rates and good retention at follow-up in a relatively young population (aged 12–16) where limited data on self-harm have previously been available. Analysis of prospective data collected over 12 months provided unique insight into predicting future self-harm. However, the self-harm measures used were general items and did not provide detail about type of self-harm, suicidal intent, or reasons behind self-harm and outcome measures were reliant on self-report. Participants who provided complete self-harm data were on average younger and less psychologically distressed at baseline than those who did not, indicating that those with more severe problems may have been under-represented. The study involved secondary analysis of the questionnaires administered in their original form, and did not directly assess the use of the items as a complete measure in screening for self-harm in community context. Further research would be required to assess the psychometric properties of a ‘new’ targeted measure of thoughts and feelings associated with self-harm, and relevant permissions from copyright holders would be required before the questionnaires could be amended in this way.

## Conclusions

The findings of this study suggest that brief assessment of negative emotion and self-esteem could be a fruitful approach in improving recognition of those at risk of self-harm in community setting. Further research should test this approach using a complete measure (subject to relevant permissions) in more diverse community contexts, and establish whether it is an acceptable and effective method when used under everyday conditions.

## Competing interests

The authors declare that they have no competing interests.

## Authors’ contributions

RP drafted the manuscript and was the trial manager for the RCT from which the data was derived. PS was the principal investigator for the project and KS led the East Midlands based research team. MS and AAM designed and carried out the statistical analysis. AM was involved with developing the original idea and rationale for the analysis. All authors have contributed to and commented on previous versions of this manuscript and have approved the final version.

## Authors’ information

RP is a health psychologist with a special interest in prevention and early intervention of chronic health problems, now based at the Wales School for Primary Care Research at Cardiff University. MS is a medical statistician and completed a National Institute for Health Research Methods Training Fellowship while she carried out the analysis for this study, working alongside AAM (Reader in Health Services Research) at Bristol University. AM is a lecturer at the University of Sheffield with a special interest in emotion and peer attachment in children and adolescents. KS is a Clinical Associate Professor and Reader in Child & Adolescent Psychiatry at Nottingham University. PS is a Professor of Child and Family Mental Health at the University of Bath where he leads the Child & Adolescent Mental Health research group. He is a consultant clinical psychologist with 30 years of experience working in child and adolescent mental health services (CAMHS).

## Pre-publication history

The pre-publication history for this paper can be accessed here:

http://www.biomedcentral.com/1471-2458/13/604/prepub

## Supplementary Material

Additional file 1 Area Under the ROC Curve (AUC), sensitivity and specificity at suggested cut-off points to detect self harm (none vs. any reported over the past six months). Click here for file
