# Supplementary material for: Could a brief assessment of negative emotions and self-esteem identify adolescents at current and future risk of self-harm in the community? A prospective cohort analysis
Source: BMC Public Health. 2013 Jun 22;13:604. doi: 10.1186/1471-2458-13-604 (PMC3733623; doi:10.1186/1471-2458-13-604)
Supplement: Additional file 1 — Area Under the ROC Curve (AUC), sensitivity and specificity at suggested cut-off points to detect self harm (none vs. any reported over the past six months). [file 1471-2458-13-604-S1.doc]

Additional file 1: Area Under the ROC Curve (AUC), sensitivity and specificity at suggested cut-off points to detect self harm (none vs. any reported over the past six months)

|  |  |  | Males |  |  |  | Females |  |
| --- | --- | --- | --- | --- | --- | --- | --- | --- |
| Baseline self-harm | AUC | Cut-off | Sensitivity  (%) | Specificity (%) | AUC | Cut-off | Sensitivity (%) | Specificity (%) |
| Personal failure | 0.76 | ≥2 | 67.26 | 78.03 | 0.84 | ≥3 | 76.11 | 79.61 |
| Physical symptoms of anxiety/ depression | 0.81 | ≥4 | 69.30 | 76.59 | 0.82 | ≥4 | 80.45 | 71.43 |
| Positive self-esteem | 0.70 | ≥3 | 76.32 | 66.71 | 0.79 | ≥6 | 75.00 | 72.43 |
| Self-harm at 6 months |  |  |  |  |  |  |  |  |
| Personal failure | 0.74 | ≥1 | 75.81 | 58.74 | 0.78 | ≥2 | 74.88 | 64.03 |
| Physical symptoms of anxiety/ depression | 0.73 | ≥3 | 68.00 | 66.40 | 0.78 | ≥4 | 74.26 | 71.39 |
| Positive self-esteem | 0.62 | N/A |  |  | 0.74 | ≥6 | 67.50 | 72.04 |
| Self-harm at 12 months |  |  |  |  |  |  |  |  |
| Personal failure | 0.65 | N/A |  |  | 0.75 | ≥2 | 73.56 | 63.97 |
| Physical symptoms of anxiety/ depression | 0.69 | N/A |  |  | 0.75 | ≥4 | 68.75 | 70.77 |
| Positive self-esteem | 0.59 | N/A |  |  | 0.71 | ≥5 | 59.80 | 71.05 |
